# Resting State Connectivity Between Medial Temporal Lobe Regions and Intrinsic Cortical Networks Predicts Performance in a Path Integration Task

**DOI:** 10.3389/fnhum.2018.00415

**Published:** 2018-10-16

**Authors:** Sarah C. Izen, Elizabeth R. Chrastil, Chantal E. Stern

**Affiliations:** ^1^Department of Psychological & Brain Sciences and Center for Memory & Brain, Boston University, Boston, MA, United States; ^2^Athinoula A. Martinos Center for Biomedical Imaging, Massachusetts General Hospital, Boston, MA, United States; ^3^Department of Geography, University of California, Santa Barbara, Santa Barbara, CA, United States

**Keywords:** resting state, navigation, path integration, default mode network, central executive network, fronto-parietal, executive function, memory

## Abstract

Humans differ in their individual navigational performance, in part because successful navigation relies on several diverse abilities. One such navigational capability is *path integration*, the updating of position and orientation during movement, typically in a sparse, landmark-free environment. This study examined the relationship between path integration abilities and functional connectivity to several canonical intrinsic brain networks. Intrinsic networks within the brain reflect past inputs and communication as well as structural architecture. Individual differences in intrinsic connectivity have been observed for common networks, suggesting that these networks can inform our understanding of individual spatial abilities. Here, we examined individual differences in intrinsic connectivity using resting state magnetic resonance imaging (rsMRI). We tested path integration ability using a loop closure task, in which participants viewed a single video of movement in a circle trajectory in a sparse environment, and then indicated whether the video ended in the same location in which it started. To examine intrinsic brain networks, participants underwent a resting state scan. We found that better performance in the loop task was associated with increased connectivity during rest between the central executive network (CEN) and posterior hippocampus, parahippocampal cortex (PHC) and entorhinal cortex. We also found that connectivity between PHC and the default mode network (DMN) during rest was associated with better loop closure performance. The results indicate that interactions between medial temporal lobe (MTL) regions and intrinsic networks that involve prefrontal cortex (PFC) are important for path integration and navigation.

## Introduction

Humans differ considerably in their individual navigational abilities, and successful navigation relies on several different skills and capabilities (Wolbers and Hegarty, 2010; Chrastil, 2013). One such navigational ability is *path integration*, the constant updating of the navigator’s position and orientation during movement, particularly in sparse environments without landmarks (Mittelstaedt and Mittelstaedt, 1980, 1982; Byrne et al., 2007). Significant individual variability has been observed in path integration abilities in human navigators (Loomis et al., 1993; Klatzky et al., 1999). Intrinsic differences between individuals in both brain structure and function could provide mechanisms that underlie these varying abilities. We previously examined structural differences, finding that better navigators in a path integration task had larger local gray matter volume in the hippocampus, retrosplenial cortex (RSC) and medial prefrontal cortex (mPFC; Chrastil et al., 2017). In the present study, we examined intrinsic functional connectivity differences using the same path integration paradigm.

The goal of this study was to examine the relationship between path integration abilities and functional connectivity to canonical intrinsic brain networks. Intrinsic networks within the brain reflect past inputs and communication (Damoiseaux et al., 2006; Fox and Raichle, 2007; Papo, 2013) as well as structural architecture (van den Heuvel et al., 2009), and have a strong relationship with task-based networks observed during functional tasks (Laird et al., 2011; Cole et al., 2014a). Individual differences in intrinsic connectivity have been observed for common networks (Mueller et al., 2013), suggesting that these networks can inform our understanding of individual spatial abilities. Here, we examined individual differences in intrinsic connectivity using resting state magnetic resonance imaging (rsMRI), in which participants were scanned at rest while maintaining fixation on a crosshair. We then tested whether functional connectivity to rsMRI networks was correlated with performance in a path integration task that they had completed earlier in the scan session.

Specifically, we were interested in intrinsic functional communication between navigation brain regions and the default mode network (DMN) and between navigation brain regions and the central executive network (CEN). The DMN and CEN were chosen *a priori* because of their involvement in and potential importance to memory and navigation. The DMN is linked to episodic memory and representations of self (Buckner and Carroll, 2007; Buckner et al., 2008; Laird et al., 2011), both of which could be important for tracking self-motion and remembering a target location. Many regions of the DMN, including the hippocampus, RSC and mPFC are also associated with activity during navigation tasks (Maguire et al., 1998; Shelton and Gabrieli, 2002; Wolbers and Büchel, 2005; Brown et al., 2010, 2016; Sherrill et al., 2013; Marchette et al., 2014; Chrastil et al., 2016). The CEN contains fronto-parietal regions, and consists of highly-connected hub regions that allow for adaptive implementation of task demands, linking this network to executive control functions (Dosenbach et al., 2006, 2007; Seeley et al., 2007; Cole et al., 2013, 2014b). Path integration requires working memory to keep track of the home location, while also updating new incoming spatial information and resisting distraction. These executive control functions could play a key role in understanding individual differences in path integration abilities.

Previous research in both animals and humans suggest that the medial temporal lobe (MTL) regions of hippocampus, parahippocampal cortex (PHC) and entorhinal cortex are likely candidates to support path integration abilities, as are RSC and mPFC. Rodent models have found several cellular fundamentals for path integration, including place cells in the hippocampus (O’Keefe and Nadel, 1978), grid cells in entorhinal cortex (Fyhn et al., 2004) and head direction cells in postsubiculum and RSC (Taube et al., 1990; Chen et al., 1994; Cho and Sharp, 2001). Functional imaging studies have demonstrated that hippocampal activity predicts accuracy in navigation in sparse environments (Wolbers et al., 2007; Sherrill et al., 2013), and PHC activity has also been observed during path integration (Sherrill et al., 2013). Lesions of the hippocampus and entorhinal cortex have been shown to cause impairments of path integration in rodents (Whishaw et al., 1997; McNaughton et al., 2006; Brun et al., 2008). BOLD activity in the hippocampus, PHC and RSC increases with Euclidean distance from the home location and with increased translation and rotation during virtual self-motion (Chrastil et al., 2015, 2016), suggesting that these regions support path integration. Together, the previous literature indicates a key role for MTL as part of a path integration network, thus, we expected functional connectivity related to MTL areas in the present study.

Path integration often involves tracking a start or home location and we previously found task-based functional imaging evidence in support of a homing signal in the human brain (Chrastil et al., 2015). We now focus on mechanisms that could underlie this homing signal. To achieve this goal, we examined individual differences in path integration performance. Understanding the relationship between path integration accuracy and network connectivity could provide insight into: (i) which brain areas contribute to path integration performance; and (ii) how those regions work in concert with other brain regions to yield accurate path integration. We predicted that better navigators would demonstrate increased functional connectivity between brain regions that support navigation, including the hippocampus, PHC, entorhinal cortex and RSC, and several canonical cortical networks. Specifically, we predicted that functional communication with the DMN (which has been linked to episodic memory and representations of self (Buckner and Carroll, 2007; Buckner et al., 2008; Laird et al., 2011)) and the CEN (linked to executive control (Dosenbach et al., 2006, 2007; Seeley et al., 2007; Cole et al., 2013, 2014b)) would be associated with path integration accuracy.

## Materials and Methods

### Participants

Thirty-one participants were recruited for this study from the Boston University community as part of previous studies (Chrastil et al., 2015, 2016). This study was carried out in accordance with the recommendations of experimental protocol guidelines, Partners Human Research Committee and the Boston University Charles River Campus Institutional Review Board. The protocol was approved by both the Partners Human Research Committee and the Boston University Charles River Campus Institutional Review Board. All subjects gave written informed consent in accordance with the Declaration of Helsinki. Because resting state analysis is particularly susceptible to movement artifacts (Van Dijk et al., 2010, 2012; Satterthwaite et al., 2013), participants with absolute movement >1 mm were eliminated from analysis to achieve the resolution necessary for network analysis. Two participants were eliminated from the final analysis due to excessive motion during resting state (rsfMRI) scanning, three participants were not scanned due to claustrophobia, one participant was found to be ineligible after screening, and one participant fell asleep during the experimental task. Twenty-four participants were included in the final data analysis (mean age 23.13 ± 4.18 (SD); 10 males, 14 females). Twenty-two of these participants were right handed, two were left handed. All participants had no history of neurological disorders.

### Stimuli and Tasks

**Figure 1:**
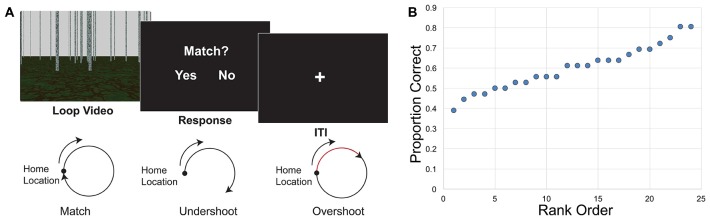
Loop closure task. **(A)** Participants watched a video of movement in a loop trajectory in a sparse, landmark-free environment. At the end of the video they decided whether the video ended in the same place in which it started (match) or somewhere else (non-match). Bottom, illustration of match and non-match trials. Both overshoots and undershoots of the home location were considered non-matches. **(B)** Behavioral results indicate the distribution of performance. Individual proportion correct of the 24 participants ranged from 0.389 to 0.806, and are displayed here in rank order from worst to best performance.

Complex path integration and self-motion processing involve tracking location, often the start or home location. This paradigm required participants to track self-motion during videos shown from a first-person perspective. Briefly, in the complex path integration task (loop closure task), participants viewed a single video of movement that traveled in a circle in a sparse environment (Figure 1) and then indicated whether the video ended in the same location in which it started (Chrastil et al., 2015). This study is based on additional analyses from our previous fMRI study on the neural correlates of path integration. A description of the loop closure task is presented here, and our publication introducing these paradigms (Chrastil et al., 2015) provides a longer description of the stimuli and task that is relevant to both the initial fMRI study and the current connectivity study.

#### Environment

The virtual environment was developed using POV-Ray v.3.6^1^, a 3D ray-tracing modeling program. The environment consisted of a textured ground plane with approximately 150 textured poles, or “trees,” randomly placed in the scene (Figure 1A). The textured ground and trees in the environment provided optic flow information during the video presentation of movement. The trees were taller than the top of the screen so that height changes could not be used as a cue to distance. The large number of trees and random placement discouraged participants from using the scene arrangement as a landmark, and each video had a different random arrangement of the trees. Movement in the videos never passed directly through a tree. Self-motion information used in this study stemmed purely from visual motion, with no vestibular or proprioceptive input, due to the constraints of fMRI scanning. Videos of movement in the environment were presented as a series of images at 30 frames per second. The videos were presented to participants using E-Prime 2.0 (Psychology Software Tools, Inc.), which also recorded the exact timing of stimulus presentation and participants’ responses.

#### Loop Closure Task

In the loop closure task, the camera movement in the video traveled in a circular pattern. Once the video ended, participants had to indicate whether the movement in the video ended at the same location in which it started, at the home location. Half of the videos ended in the home location (“match,” a full 360° traversal of the loop), and half were non-matches, ending at another point along the circle. Half of the non-matches were undershoots, such that the movement only traversed partway around the circle (225° of the loop). The other half were overshoots, such that movement went past the home location and went partway around a second loop (495° of the loop). Participants were given clear instructions that overshoots were considered non-matches, and that it was important to determine whether the end point itself was the same as the start location. Three different radii of curvature (2.0, 3.0 and 4.5 virtual units) and two different travel speeds (1.5 and 2.0 virtual units/s) were used in the loop task, crossed to yield six angular speeds (0.33, 0.44, 0.50, 0.67, 0.75 and 1.00 radians/s). The length of the videos for the loop task ranged between approximately 4–25 s, with an average of 11.5 s. After the video, a response screen was presented, and participants had up to 2 s to respond whether the loop returned to the home location. A 6 s intertrial interval (ITI) began as soon as the response was recorded, thus the duration of the response was based on participants’ reaction time. Loops turned both to the right and to the left in equal numbers; we combined over left and right turning direction for analysis.

#### Resting State Task

The functional imaging of interest took place during a resting state scan that occurred after the test runs of the path integration task. During the resting state scan, participants were instructed to keep their eyes open and look at a fixation cross, but they could think about whatever they liked. One 6:12 min long resting state scan was acquired after the experimental task scan runs.

### Procedure

#### Pre-scan Training

Participants were trained outside the scanner the day prior to scanning. Participants were given a general description of movement in the environment and shown a short example. In addition to the loop closure task, participants were trained on additional tasks not presented here (loop, distance, angle, curve and static image change; see Chrastil et al., 2016). They were then given specific instructions and several practice runs with feedback for each of the tasks in turn. Participants also completed several individual abilities questionnaires, which are discussed in detail elsewhere (Chrastil et al., 2015, 2016, 2017).

#### Experimental Task

While the structural scans were being acquired, participants were given a practice run with feedback using examples from the training, with eight trials per task block. Following practice, there were six functional test runs, randomized across participants, for a total of 36 trials per condition. Each of the test runs consisted of one block each of the experimental tasks (loop, distance, angle, curve and static image mentioned in the section on pre-scan training). Each block contained six trials of the task, with match and non-match trials counterbalanced across runs. The task order of each block was counterbalanced across runs. Length and direction of movement, as well as speed of travel, were counterbalanced across conditions and runs. Because the ITI began as soon as participants made their responses, the scan time for each of the six runs varied somewhat, but generally lasted just under 10 min. Total scan time for the experimental task was approximately 1 h. Following the experimental task runs, the 6:12 min resting-state scan was acquired.

### MRI Image Acquisition

Images were acquired at the Athinoula A. Martinos Center for Biomedical Imaging, Massachusetts General Hospital in Charlestown, MA, USA using a 3 Tesla Siemens MAGNETOM TrioTim scanner with a 32-channel Tim Matrix head coil. High-resolution T1-weighted multi-planar rapidly acquired gradient echo (MP-RAGE) structural scans were acquired using Generalized Autocalibrating Partially Parallel Acquisitions (GRAPPA; TR = 2530 ms; TE = 3.31 ms; flip angle = 7°; slices = 176; resolution = 1 mm isotropic). T2*-weighted BOLD images were acquired for the resting state scan using an echo planar imaging (EPI) sequence (TR = 2,000 ms; TE = 30 ms; flip angle = 85°; slices = 33, resolution = 3.0 × 3.0 × 3.44 mm, interslice gap of 0.5 mm). Functional image slices were aligned parallel to the long axis of the hippocampus.

### Behavioral Analysis

The primary outcome measure of path integration ability was the proportion of correct trials. Behavioral performance was assessed using MatLab (MathWorks) and SPSS20 (IBM). A one-sample *t*-test was used to assess overall performance against chance levels (0.50 proportion correct).

### fMRI Preprocessing

Resting state BOLD images were reoriented in SPM8 (Statistical Parametric Mapping, Wellcome Department of Cognitive Neurology, London) so that the origin (coordinate x, y, z = [0, 0, 0]) was the anterior commissure. The remainder of the preprocessing was done with FSL (FMRIB, Oxford, UK; FSL version 5.0.6) using the MELODIC preprocessing stream (Jenkinson et al., 2012). We used the FSL default settings unless otherwise noted. Brain extraction was done using BET to isolate the brain from the skull and other surface features (Smith, 2002) and the first five volumes were deleted. MCFLIRT was performed for motion correction (Jenkinson et al., 2002), and participants were removed from the analysis if absolute mean displacement exceeded 1 mm. Spatial smoothing with a Gaussian kernel of full-width half-maximum (FWHM) of 6 mm was performed, along with a high-pass filter with sigma set at the default 100 s. FLIRT was used to register functional images both to their own MPRAGE image and to MNI standard space (Montreal Neurological Institute, Montreal, QC, Canada; Jenkinson and Smith, 2001; Jenkinson et al., 2002). In order to remove any signal representing noise, each participant’s individual components were visually inspected and artifacts were removed using the *fsl_regfilt* command line tool.

### Functional Connectivity Analysis

Functional connectivity analysis was used to uncover the relationship between performance on the loop closure task and network connectivity. The regression analysis correlated performance with the strength of network connectivity. The significant effects shown in each voxel in the results indicate connectivity with the network of interest that varied by performance at that voxel. We conducted a whole-brain analysis of this question. Thus, this analysis tests whether the strength of connectivity between any given voxel in the brain and the CEN or DMN increased with accuracy in the loop task.

#### Network Definitions

**Figure 2:**
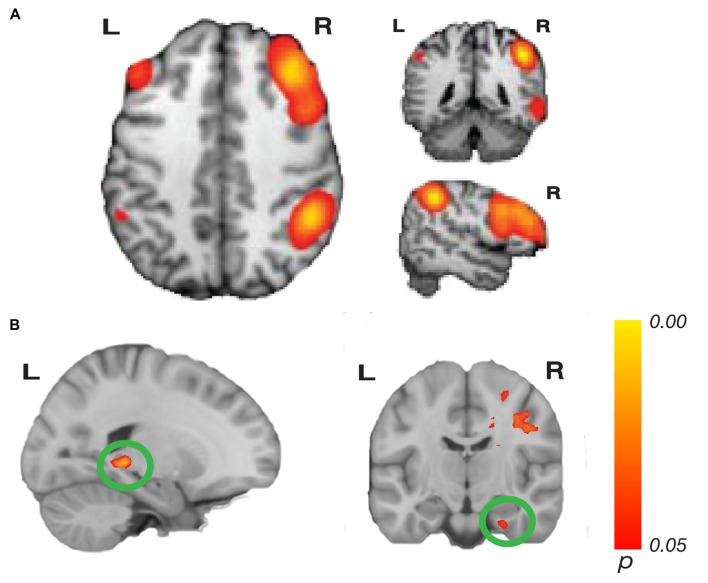
Network connectivity results of the central executive network (CEN). **(A)** The CEN as defined by Laird et al. (2011; modified with permission). **(B)** Activations show regions where resting state connectivity to previously defined template networks was significantly associated with accuracy (Whole-brain analysis, threshold-free cluster enhancement (TFCE) with permutation testing, family-wise *p* < 0.05). Hippocampus tail (left; xyz: −20, −38, −2) and entorhinal cortex (right; xyz: 28, −14, −32) connectivity to the right CEN increased with path integration accuracy. Complete results for the right CEN are shown in Table 1.

**Figure 3:**
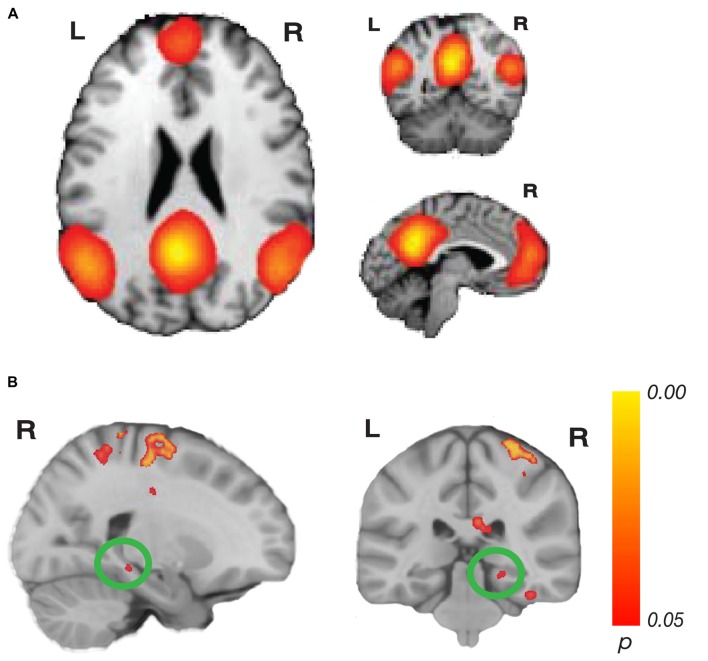
Network connectivity results of the default mode network (DMN). **(A)** The DMN as defined by Laird et al. (2011; modified with permission). **(B)** Activations show regions where resting state connectivity to previously defined template networks was significantly associated with accuracy (Whole-brain analysis, TFCE with permutation testing, family-wise *p* < 0.05). Parahippocampal cortex (PHC; xyz: 22, −32, −10) connectivity to the DMN increased with accuracy. Complete results for the DMN are shown in Table 2.

BrainMap 20 templates (Filippini et al., 2009; Laird et al., 2011) are pre-defined templates of 20 major intrinsic cortical networks. We used these templates to test connectivity to three networks: the CEN, containing fronto-parietal regions, and the DMN, containing the medial prefrontal and posterior cingulate/precuneus areas. The CEN is separated into two networks in the BrainMap 20 templates, with one network dedicated to the right hemisphere, and one dedicated to the left hemisphere network, yielding three total networks of interest. These networks were chosen *a priori* because of their involvement in and potential importance to memory and navigation (Seeley et al., 2007; Buckner et al., 2008; Cole et al., 2013). The loop closure task requires encoding distance and orientation during movement, while monitoring the home location. This process requires working memory to track and update the home location during movement, resistance to distraction from internal and external stimuli, rapid processing of incoming visual information and tracking of path integration errors, which could relate to the CEN. Episodic memory, and thus the DMN, could be important for performance of the loop closure task because the participant needs to create a memory of the target location and continuously update their location in space. These networks were predefined in the FSL templates (Figures 2A, 3A), which included all brain regions in the network. Each complete network was the target of a whole-brain analysis to test for areas that showed significantly increasing connectivity to that network as a function of accuracy in the task.

#### Regression Analysis

Dual regression was performed using the pre-defined BrainMap 20 templates (Filippini et al., 2009; Laird et al., 2011) to identify regions of the brain that were functionally connected to each network. In dual-regression, first a subject-specific timeseries was generated by regressing group-level spatial maps (i.e., the BrainMap template for a given network) as spatial regressors into each individual subjects’ 4D resting state dataset. Subsequently, those subject-specific timecourses were regressed into the same 4D resting-state dataset as temporal regressors to get one subject-specific spatial map of the connectivity to that network (Nickerson et al., 2017). We then tested for individual differences by including accuracy in the loop closure task as the primary regressor of interest. We included sex and age as covariates in the model to control for these potential confounding factors. We conducted a one-sample *t*-test for each regressor, examining the relationship between accuracy in each of the behavioral task and connectivity to the *a priori* networks of interest. We examined both positive (related to better performance) and negative (related to poorer performance) correlations.

We note that our results could show regions both outside of the network of interest and regions within the network that were significantly connected related to performance because our whole-brain analysis examines all voxels in the brain. For example, the RSC is part of the DMN, and a significant finding in RSC in the DMN contrast would indicate that RSC has significantly greater connectivity to other parts of the DMN in people who did better at the task. Thus, some of our results could be within-network, although they are not explicitly stated as such.

To conduct this whole-brain analysis for significant connectivity to the three complete networks that was related to accuracy in the loop closure task, we used *randomize*, a permutation testing method, to test for significance. We conducted 500 random draws of the data, and then compared our model with these random permutations. Dual regression and *randomize* were run using threshold-free cluster enhancement (TFCE), correcting for family-wise error to a level of *p* < 0.05. TFCE is a method that does not require a cluster-forming threshold and has been shown to give better sensitivity (Smith and Nichols, 2009), such that smaller but very strong clusters were permitted, rather than weaker but larger cluster extents, which can make localization difficult. Thus, the mass of significant clusters passed the permutation test threshold of corrected *p* < 0.05. In addition to this correction, we excluded clusters with five or fewer voxels from the results. We used Damasio (Damasio, 2005) and Pruessner (Pruessner et al., 2000, 2002) as references for localization in the cortex.

## Results

### Behavioral Results

Behavioral performance has been described in depth elsewhere (Chrastil et al., 2015, 2017), but key findings that relate to this analysis are repeated here. Overall proportion correct in the loop closure task was 0.600 (SEM ± 0.023). Performance was significantly higher than chance (0.5) performance (*t*_(23)_ = 4.366, *p* < 0.001). Individual proportion correct ranged from 0.389 to 0.806, with a fairly even distribution of performance (Figure 1B), suggesting that the correlations with connectivity were not driven by outliers.

### Network Connectivity Results

We analyzed resting state connectivity using previously defined networks, testing whether the strength of connectivity to these networks increased with accuracy in the loop task. We examined the relationship between accuracy in the loop closure task and connectivity to three *a priori* networks of interest—the right and left CENs and the DMN. A significant result in each cluster shows that the strength of connectivity between voxels in that cluster and the CEN or DMN increased with accuracy in the loop task. Importantly, variations in connection strength to these networks could occur *within* regions of the network itself as well as brain regions *outside* of the network. Here, we report MNI x, y, z coordinates of peak voxels in each cluster, as well as the *t*- and corrected *p*-values for the peak voxel, and the size of the cluster (k).

Our whole-brain analysis looked for areas that showed increasing connectivity to a network as a function of accuracy in the path integration task. This analysis revealed significant intrinsic connectivity between the right CEN and the left hippocampus tail (xyz: −20, −38, −2; *t*_(23)_ = 6.78; *p* = 0.016; *k* = 87) and right entorhinal cortex (xyz: 28, −14, −32; *t*_(23)_ = 5.40; *p* = 0.04; *k* = 20; Figure 2) that was related to accuracy in the loop task. In addition, a large cluster (*k* = 3275) was found in the right hemisphere, which included a large swath of white matter but also extended into PHC (xyz: 18, −30, −10; *t*_(23)_ = 6.16; *p* = 0.038) as well as thalamus, caudate and cingulate. In addition to these clusters, we found a cluster that spanned middle temporal gyrus and superior temporal sulcus, a cluster in cingulate sulcus, and two clusters in the cerebellum (one cluster spanned left and right cerebellum). Table 1 has complete results of the right CEN results. There was no significant performance-related connectivity to the left CEN, and no significant relationship with worse performance.

**Table 1:** Brain regions where greater accuracy in the path integration task was associated with increased connectivity to the right central executive network (CEN).

Cluster size (k)	Brain region	*p*-value	Left MNI *x*, *y*, *z*	*p*-value	Right MNI *x*, *y*, *z*
3275	White matter extending into			0.036	28, −68, 6
	Thalamus			0.034	16, −28, 8
	Caudate			0.04	18, 6, 18
	Cingulate			0.02	14, −26, 32
	Parahippocampal Cortex			0.038	18, −30, −10
87	Hippocampus Tail	0.016	−20, −38, −2		
56	Middle Temporal Gyrus/Superior Temporal Sulcus	0.024	−52, −32, −8		
39	Cerebellum	0.032	−2, −56, −4	0.04	2, −56, −4
20	Entorhinal Cortex			0.04	28, −14, −32
7	Cingulate Sulcus			0.048	12, 14, 38
7	Cerebellum	0.048	−8, −48, −14		

*Here, we report MNI x, y, z coordinates of peak voxels in each cluster, as well as the *t*- and corrected *p*-values for the peak voxel, and the cluster size (k)*.

For the DMN, we found a significant relationship related to accuracy with PHC (xyz: 22, −32, −10; *t*_(23)_ = 6.05; *p* = 0.044; *k* = 14; Figure 3). This cluster borders on the hippocampus and subiculum region. An additional cluster in the MTL region included the collateral sulcus and part of the parahippocampal gyrus (xyz: −32, −28, −24; *t*_(23)_ = 5.46; *p* = 0.034; *k* = 102). Other regions found in the DMN analysis included a cluster spanning pre-central gyrus, postcentral gyrus and superior parietal lobule, a cluster in precuneus, two clusters in the cerebellum, a cluster in cingulate sulcus, several clusters in temporo-occipital gyrus and a cluster in superior temporal sulcus. No significant results for the negative contrast were found. Complete results for the DMN can be found in Table 2.

**Table 2:** Brain regions where greater accuracy in the path integration task was associated with increased connectivity to the default mode network (DMN).

Cluster size (k)	Brain region	*p*-value	Left MNI *x*, *y*, *z*	*p*-value	Right MNI *x*, *y*, *z*
1838	Precentral Gyrus			0.016	30, −18, 64
	Postcentral Gyrus			0.01	30, −38, 64
	Superior Parietal Lobule			0.044	30, −54, 68
123	Precuneus	0.026	−6, −54, 56		
102	Collateral Sulcus	0.034	−32, −28, −24		
88	Cingulate Sulcus	0.03	−18, −26, 38		
81	Temporo-Occipital Gyrus			0.034	40, −32, −24
72	Temporo-Occipital Gyrus	0.044	−32, −6, −44		
69	Inferior Temporal Gyrus	0.046	−48, −10, −36		
59	Cerebellum	0.044	−24, −46, −26		
32	Superior Temproal Sulcus			0.044	46, −20, −10
31	Cerebellum	0.048	−2, −58, −22		
22	Precentral Gyrus			0.046	60, −2, 34
19	Temporo-Occipital Gyrus			0.048	34, −18, −34
14	Parahippocampal Cortex			0.044	22, −32, −10
10	Temporo-Occipital Gyrus	0.048	−38, −34, −20		

*Here, we report MNI x, y, z coordinates of peak voxels in each cluster, as well as the *t*- and corrected *p*-values for the peak voxel, and the cluster size (k)*.

## Discussion

In this experiment, we combined behavioral accuracy in a loop closure task, which provided a measurement of path integration ability, and resting state fMRI analysis (rsMRI). We found that better performance in the loop closure task was associated with increased functional connectivity between the right CEN and hippocampus tail, PHC and entorhinal cortex. We also found that functional connectivity between the DMN and PHC was associated with better loop closure task performance. The results suggest that interactions between MTL regions and both the CEN and DMN are important for navigation. In particular, both CEN and DMN have major network nodes in PFC, indicating a link between individual navigational abilities and executive function, working memory and episodic memory processes.

Our first major finding is that increased intrinsic connectivity between MTL regions and the right CEN is predictive of navigational ability. The CEN is important for adaptive implementation of shifting task demands and other executive control functions (Dosenbach et al., 2006; Seeley et al., 2007; Cole et al., 2013, 2014b). Executive control could be important for the loop closure task because performance of the task requires working memory to track and update the home location during movement, resistance to distraction from internal and external stimuli, rapid processing of incoming visual information and tracking of path integration errors. BOLD activation has previously been observed during other navigational tasks in nodes of the CEN, including dorso- and ventro-lateral PFC (dlPFC and vlPFC), posterior parietal cortex and intraparietal sulcus (IPS; Spiers and Maguire, 2006; Brown et al., 2010; Sherrill et al., 2013; Howard et al., 2014; Chrastil et al., 2016; Javadi et al., 2017). During our functional imaging of the loop closure task, we found parietal BOLD activation in regions that are part of the CEN during correct loop closure trials (Chrastil et al., 2015). Together, these findings indicate a role for this fronto-parietal network during path integration and navigation.

Surprisingly, we did not find any significant connectivity with the left CEN that was related to accuracy in the loop closure task. It is possible that the left networks connected equally well to all navigators, or that lateralization of this network plays a significant role. Although the left hemisphere has generally been more closely associated with executive functioning, the right hemisphere tends to be more associated with spatial processing (e.g., Smith and Jonides, 1999; Carpenter et al., 2000; Duncan and Owen, 2000). This divergence could underlie our finding significant connectivity for only the right lateralized CEN.

The CEN showed intrinsic connectivity with several navigational brain regions. Specifically, we found increased connectivity between regions within the right CEN and the hippocampus, entorhinal cortex and PHC in better navigators. These MTL regions are vital to path integration, and experiments in both animals and humans, as well as computational models, have demonstrated that these areas are important for the updating of spatial location. Grid cells in rodent entorhinal cortex demonstrate firing patterns that code spatial arrays, facilitating the updating of spatial location (Fyhn et al., 2004; Hafting et al., 2005). The spatial information in grid cells could then be used to update location information in hippocampal place cells (O’Keefe and Nadel, 1978; O’Keefe and Burgess, 1996; Burgess et al., 2007; Hasselmo, 2009). These grid and place cell-like firing patterns have also been observed in humans (Ekstrom et al., 2003; Doeller et al., 2010; Jacobs et al., 2013), suggesting a similar system for path integration. Entorhinal cortex also codes for direction and distance to goals in humans and has larger gray matter volume in better navigators (Howard et al., 2014; Chadwick et al., 2015; Sherrill et al., 2018), while the hippocampus has been shown to be important for path integration in a number of studies (Philbeck et al., 2004; Wolbers et al., 2007; Morgan et al., 2011; Sherrill et al., 2013; Howard et al., 2014; Yamamoto et al., 2014; Chrastil et al., 2015; but see also, Shrager et al., 2008). These functional findings, together with the results presented here, suggest that communication between brain regions important for path integration and executive function areas is important for successful navigation.

Our second major finding was that better navigators have increased intrinsic connectivity between PHC and the DMN. Although the DMN was originally viewed as a task-negative network, it has since been linked to many cognitive processes, including episodic memory and representations of oneself (Buckner and Carroll, 2007; Buckner et al., 2008; Laird et al., 2011). Episodic memory could be important for performance of the loop closure task because the participant needs to create a memory of the target location and continuously update their location in space. Self-referential processing could also be vital to tracking self-motion during loop closure and visualizing the path during movement. A recent study found cooperative interactions between the DMN, the right CEN and the mPFC during an internally-directed memory search task (Kragel and Polyn, 2015), suggesting that the networks we identified here are important for a broad variety of memory tasks, especially those related to self-processing.

Many of the regions commonly observed in navigation tasks are hubs of the DMN (Maguire et al., 1998; Shelton and Gabrieli, 2002; Wolbers and Büchel, 2005; Brown et al., 2010; Sherrill et al., 2013; Marchette et al., 2014), and in the functional version of this task we found corresponding BOLD activation in many DMN regions, including the hippocampus, RSC, PHC and angular gyrus (Chrastil et al., 2015). In the present study, we found PHC in particular to be related to DMN activity; this part of PHC borders on the hippocampus and subiculum region, an area known for grid cells, head direction cells and boundary vector cells (Taube et al., 1990; Lever et al., 2009; Boccara et al., 2010; Vass and Epstein, 2013). PHC has also been shown to be relevant to spatial context and scene processing (Bar and Aminoff, 2003; Davachi et al., 2003; Epstein, 2008; Epstein and Vass, 2013; Preston and Eichenbaum, 2013; Brown and Stern, 2014), and as well as to path integration tasks (Sherrill et al., 2013; Chrastil et al., 2015, 2016). Together, previous research on PHC suggests a strong role for processing self-motion during path integration by means of updating spatial information. The results of the present study are consistent with these findings, and suggest that better navigators have increased ability to process the incoming spatial information to update their self-localization in the environment.

Regions of the PFC are nodes in both the CEN and DMN. Dorsal mPFC, dlPFC and vlPFC are nodes in the CEN (Seeley et al., 2007; Cole et al., 2013), and ventral mPFC is a node in the DMN (Buckner et al., 2008). Previous research indicates that executive function, working memory, cognitive control and goal-directed behavior are important parts of successful navigation (Spiers, 2008). In this study, we found that good navigators have functional communication between navigation regions in the MTL and the CEN. However, it is possible that the connections with prefrontal networks observed here are driven by completely independent network associations; our correlational analysis cannot determine the direction of causality or a potential independent source. Researchers must look beyond the MTL, including potential links with prefrontal function, to fully understand the neural mechanisms underlying spatial navigation. The strong connectivity between the MTL and the CEN as well as the DMN indicate that PFC provides a potential avenue for future research on navigational abilities.

Notably, we did not observe any connectivity effects involving RSC or mPFC, regions in which we previously found structural variation corresponding to individual path integration ability on this same task (Chrastil et al., 2017). These regions are also nodes of the DMN (Buckner et al., 2008; Andrews-Hanna et al., 2010). RSC BOLD activity has been related to tracking heading direction (Baumann and Mattingley, 2010; Marchette et al., 2014; Shine et al., 2016) and path integration (Sherrill et al., 2013; Chrastil et al., 2015, 2016). Furthermore, lesions to RSC cause impairments in path integration (Save et al., 2001; Save and Poucet, 2009). mPFC BOLD activity has been observed during path integration, both while tracking locations and while encoding the basic translations and rotations of self-motion (Spiers and Maguire, 2007; Wolbers et al., 2007; Sherrill et al., 2013; Arnold et al., 2014; Chrastil et al., 2015), suggesting that mPFC could contribute to the encoding and maintenance of spatial information during path integration. However, the lack of functional connectivity findings in the present study suggests that both RSC and mPFC communicate with the CEN and other parts of the DMN similarly across all ability levels. Although the pattern of functional connectivity may not differ, the increased gray matter volume could still impart an advantage in better navigators (Chrastil et al., 2017).

We found other notable differences between our previous structural results (Chrastil et al., 2017) and the functional connectivity analyses presented here. For example, the increased hippocampal connectivity with the CEN in the present study was found within the posterior hippocampus for better navigators, whereas structurally we previously reported larger gray matter volumes in anterior hippocampus for better navigators (Chrastil et al., 2017). Our previous gray matter volume analysis also did not uncover structural differences in either PHC or entorhinal cortex, whereas the connectivity results suggest increased connectivity in both these areas for better navigators. Together, these differences highlight the importance of conducting multiple types of analyses for a complete understanding of individual differences. In addition, these results suggest that gray matter volume and functional connectivity measurements might tap into different aspects of individual abilities. Gray matter volume could be related to intrinsic neural resources, while rsMRI could be measuring the way in which neural resources interact. Taken together, the results of the two studies indicate that people who are better at path integration have larger gray matter volume in the anterior hippocampus, RSC and mPFC, and have greater functional communication between the hippocampus tail, PHC and entorhinal cortex with the CEN, and between PHC and the DMN.

Finally, we should note some limitations for our study. Although there was substantial variation in behavioral performance, the sample size was limited. The sample size could reduce our power to distinguish true effects. In addition, the resting state scan was completed after the task, which could influence resting state function (Waites et al., 2005; Barnes et al., 2009). Thus, resting state in this case could potentially be considered another measure related to the task. Because we were measuring individual performance, the influence of task could increase the size of our effects. However, participants completed other four tasks during the course of the scan (see “Materials and Methods,” section), none of which were correlated with each other in behavioral performance (Chrastil et al., 2017), reducing potential task carry-over effects specific to loop closure.

In conclusion, we found evidence for functional communication between brain regions in the MTL that are vital for navigation and both the CEN and DMN, two cortical networks that are important for memory, self-referential processing and executive function. Individuals with greater communication between MTL regions and both the CEN and DMN had greater accuracy in the loop closure task. These results suggest that the strength of communication between navigation regions and primary memory and executive function networks is important for successful navigation. The results of this study suggest that in the future a broader examination into working memory and executive functions will be necessary to understand the breadth of human navigational abilities.

## Author Contributions

EC and SI contributed equally to this work. SI conducted the analysis and wrote the article. EC designed the research, wrote the article, collected data, and conducted analysis. CS designed the research and wrote the article.

## Conflict of Interest Statement

The authors declare that the research was conducted in the absence of any commercial or financial relationships that could be construed as a potential conflict of interest.

## Footnotes

^1^http://www.povray.org/
